# Synergistic Effect between Amoxicillin and TLR Ligands on Dendritic Cells from Amoxicillin-Delayed Allergic Patients

**DOI:** 10.1371/journal.pone.0074198

**Published:** 2013-09-16

**Authors:** Maria J. Sanchez-Quintero, Maria J. Torres, Ana B. Blazquez, Enrique Gómez, Tahia D. Fernandez, Inmaculada Doña, Adriana Ariza, Inmaculada Andreu, Lidia Melendez, Miguel Blanca, Cristobalina Mayorga

**Affiliations:** 1 Research Laboratory, Carlos Haya Hospital-IBIMA, Málaga, Spain; 2 Allergy Service Carlos Haya Hospital-IBIMA, Málaga, Spain; 3 Joint Research Unit IIS La Fe and Polytechnical University of Valencia, Spain; 4 Institute of Chemical Technology UPV-CSIC, Polytechnical University of Valencia, Valencia, Spain; Universidade Federal do Rio de Janeiro, Brazil

## Abstract

Amoxicillin, a low-molecular-weight compound, is able to interact with dendritic cells inducing semi-maturation in vitro. Specific antigens and TLR ligands can synergistically interact with dendritic cells (DC), leading to complete maturation and more efficient T-cell stimulation. The aim of the study was to evaluate the synergistic effect of amoxicillin and the TLR2, 4 and 7/8 agonists (PAM, LPS and R848, respectively) in TLR expression, DC maturation and specific T-cell response in patients with delayed-type hypersensitivity (DTH) reactions to amoxicillin. Monocyte-derived DC from 15 patients with DTH to amoxicillin and 15 controls were cultured with amoxicillin in the presence or absence of TLR2, 4 and 7/8 agonists (PAM, LPS and R848, respectively). We studied TLR1-9 gene expression by RT-qPCR, and DC maturation, lymphocyte proliferation and cytokine production by flow cytometry. DC from both patients and controls expressed all TLRs except TLR9. The amoxicillin plus TLR2/4 or TLR7/8 ligands showed significant differences, mainly in patients: AX+PAM+LPS induced a decrease in TLR2 and AX+R848 in TLR2, 4, 7 and 8 mRNA levels. AX+PAM+LPS significantly increased the percentage of maturation in patients (75%) vs. controls (40%) (p=0.036) and T-cell proliferation (80.7% vs. 27.3% of cases; p=0.001). Moreover, the combinations AX+PAM+LPS and AX+R848 produced a significant increase in IL-12p70 during both DC maturation and T-cell proliferation. These results indicate that in amoxicillin-induced maculopapular exanthema, the presence of different TLR agonists could be critical for the induction of the innate and adaptive immune responses and this should be taken into account when evaluating allergic reactions to these drugs.

## Introduction

Delayed-type hypersensitivity (DTH) reactions induced by drugs appear hours or days after the drug administration and usually affect the skin. Maculopapular exanthema (MPE), the most frequent manifestation, can be induced by different drugs, among which betalactam-induced MPE is the most widely studied [1,2]. MPE reactions are T-cell mediated with a Th1 pattern [3,4], although several studies have reported the involvement of dendritic cells (DCs) and natural killer cells in the early stages of the immune response [5,6].

DCs are professional antigen-presenting cells (APC) [7] that have a key role in the induction of specific immune responses, depending on the state of maturation at which they present the antigen to T lymphocytes [8,9]. Monocyte-derived DCs (moDCs) can be activated by allergens [10] and low-molecular-weight compounds such as nickel and drugs [11-13], thus determining tolerance versus hypersensitivity responses. We have previously demonstrated that amoxicillin (AX) induced DC maturation and an increase in lymphocyte proliferation in AX-allergic patients [14]. However, the low expression of costimulatory molecules on DCs, the mixed T-cell phenotype and the low cytokine production by both DCs and T cells suggested that AX induces, at least *in vitro*, semi-maturation on moDCs, a possibility that is consistent with other studies [9]. This may be explained by the fact that other factors could be present during the acute allergic reactions which are absent in the *in vitro* system. These factors may be pathogen associated molecular patterns (PAMPS) that interact with Toll-like receptors (TLRs) on DCs [9]. Ten TLRs have been described in humans and they can be divided into extracellular (TLR1, TLR2, TLR4, TLR5, and TLR6) and intracellular (TLR3, TLR7, TLR8, and TLR9) [15]. Plasmacytoid and myeloid DC subpopulations show different TLR patterns, related with different functions [16]. In the case of moDCs, the expression has been shown of all TLRs except TLR7 and 9, although some degree of controversy exists [17,18].

It has also been demonstrated that specific antigens and different TLRs can synergistically interact with DCs to induce a more efficient T-cell stimulation leading to an increase in the proinflammatory response as well as T-cell polarization [19,20]. In this sense, interesting data have been published about TLR2 and 4 in hapten-induced T-cell responses, as in contact dermatitis [21]. However, for TLR7 and TLR8, although data demonstrate an important effect on DC maturation and production of proinflammatory cytokines, the role of these TLRs in drug-induced DTH has not been well characterized.

Our hypothesis in this study was that TLRs can act as a cofactor for the induction of a T-cell mediated reaction such as drug-induced MPE. To address this hypothesis, we analyzed whether the combination of AX with TLR agonists (TLR2, 4, 7 or 8) can affect the expression of TLRs on DCs, amplify DC maturation, and trigger a specific T-cell proliferation mimicking the *in vivo* response in patients with exanthematous reactions induced by AX.

## Materials and Methods

### Ethics Statement

The institutional review board “Comisión de Ética y de Investigación del Hospital Regional Universitario Carlos Haya” approved the study (#04/2009). These studies were carried out in accordance with the Declaration of Helsinki. Oral and written informed consent for all the diagnostic procedures was obtained from patients and controls.

### Patients and controls

The study involved patients with AX-induced MPE. MPE is an exanthematous reaction, with erythematous macula or papula often symmetric and typically polymorphous. Diagnosis was confirmed by a positive delayed intracutaneous test or drug provocation test (DPT) to AX, following the European Network for Drug Allergy (ENDA) guidelines [22]. Controls comprised sex- and age-matched subjects with no history of allergic reactions to any penicillin, skin-test negative to AX and confirmed tolerance to AX. Samples from patients and controls were processed following current procedures and frozen immediately after their reception and kindly provided by the BBSSPA, unit Carlos Haya Hospital.

### Generation of monocyte-derived DC

Monocytes from allergic subjects and controls were purified from peripheral blood mononuclear cells (PBMC) with anti-CD14 microbeads following the manufacturer’s protocol (Miltenyi Biotec, Bergisch Gladbach, Germany). The CD14^-^ fraction was frozen for a later lymphocyte transformation test (LTT).

CD14^+^ cells were differentiated into immature DCs (imDCs) by incubating with rhGM-CSF (200ng/mL) and rhIL-4 (100ng/mL) (both from R&D Systems Inc, Minneapolis MN) for 5 days at 37°C [14].

### DC maturation assays

imDCs (2x10^5^cells/well) were stimulated with either AX (250µg/ml) (Clamoxyl®, GlaxoSmithKline, Madrid, Spain), or TLR2 ligand, Pam3CSK4 (PAM) (5µg/ml) (InvivoGen, San Diego, CA), TLR7/8 ligand, Resiquimod (R848) (10µg/ml) (InvivoGen), or TLR4 ligand, LPS (100ng/ml) (Sigma Aldrich, St Louis, MO), alone or together. Cytokine production was measured in the culture supernatants and the cells were harvested for the following experiments:

#### DC maturation

DCs from different conditions were incubated with monoclonal antibodies (CD80, CD83, HLA-DR, CD86) (BD Pharmigen, San Diego, CA) and acquired in a FACSCanto II (BD Biosciences, Milpitas CA). Data were analyzed with FACSDiva software (BD Biosciences) and expressed as percentage of cells expressing the different markers [14]. Results were considered positive when the ratio between the percentage of stimulated cells compared with non-stimulated cells was greater than 2.

#### TLR expression on DCs

This was measured by quantitative real-time RT-PCR. RNA was extracted from DCs using the RNeasy Micro Kit (QIAGEN, Hilden, Germany) following the manufacturer’s instructions. RT was performed using Superscript II enzyme (Invitrogen, Barcelona, Spain) and oligo dT 12-18 Primer (Invitrogen, Barcelona, Spain) in a final volume of 20 mL following the manufacturer’s instructions. Synthesized cDNA was stored at -80°C until used for quantitative real-time PCR. cDNA was amplified in a Mastercycler gradient (Eppendorf, Hamburg, Germany) using SYBR green master mix and primers (both from Invitrogen; see Table 1) and in duplicate for each sample. PCR was run for 40 cycles consisting of 15 s at 95°C and 1 min at 60°C. Data were expressed as fold increase compared with levels measured in controls by using the ΔΔCt method and β-actin as a reference gene.

**Table 1:** Primer sequences to quantify the TLR expression on DC after different stimuli.

**Primer’s Name**	**Forward (5´–3´**)	**Reverse (5´–3´**)
TLR1	5´ GGGTCAGCTGGACTTCAGAG 3´	5´AAAATCCAAATGCAGGAACG3´
TLR2	5´ TGTGACCGCAATGGTATCTG 3´	5´TGTTGTTGGACAGGTCAAGG3´
TLR3	5´ CCGCCAACTTCACAAGGTAT 3´	5´AGCTCATTGTGCTGGAAATT3´
TLR4	5´ TCCATAAAAGCCGAAAGGTG 3´	5´ GATACCAGCACGACTGCTCA 3´
TLR5	5´ GGTCCATGATTCTGCGTTCT 3´	5´ GCATTGTTTCTGTGGCAAA 3´
TLR6	5´ GGAGTGATGATGGGAGGAGA 3´	5´ GGCCGAAACTGGTTTATTGA 3´
TLR7	5´ GCTCTGTGGGAGTTCTGTCC 3´	5´ ACCGTTTCCTTGAACACCTG 3´
TLR8	5´TGAAGCACATCCCAAATGAA3´	5´GCAACTCGAGACGAGGAAAC3´
TLR9	5´ CCTATTCATGGACGGCAACT 3´	5´ GAGTGACAGGTGGGTGAGGT 3´
MyD88	5´ GCACATGGGCACATACAGAC 3´	5´ TAGCTGTTCCTGGGAGCTGT 3´
β-actin	5´ GATGAGATTGGCATGGCTTT 3´	5´ CACCTTCACCGTTCCAGTTT 3´.

### Lymphocyte proliferation

CFSE (Invitrogen)-labeled autologous lymphocytes (1.5x10^5^) were co-cultured with 1.5x10^4^ imDCs as APC, in the presence of AX (250µg/ml), LPS (100ng/mL), PAM (5µg/mL) and R848 (10µg/mL), alone or in combination. After 7 days the percentage of CD3^+^ expressing CFSE^low^ was assessed by flow cytometry. The results were considered positive when the stimulation index (SI), calculated as:

$$SI=\frac{\left[\%CFSE{\text{​}}^{low}stimulated\left(ly+DC\right)\right]-\left[\%CFSE{\text{​}}^{low}unstimulated\left(ly+DC\right)\right]}{\%CFSE{\text{​}}^{low}\left(ly\right)}$$

was greater than 3.

### Cytokine production analysis

Th1/Th2 cytokine production was measured by FlowCytomix kit (Bender MedSystems, Vienna, Austria) in the culture supernatants from both DC maturation and LTT after 72h and 7 days respectively. After each stimulus, the cytokine production, expressed in pg/ml, was normalized after subtracting its respective production in imDC.

### Statistical analysis

Comparisons of the results with the different stimuli were made by chi-square (χ^2^) analysis for the qualitative variables, and for the quantitative variables comparisons were made using the non-parametric Kruskal-Wallis and Mann-Whitney tests for non-related samples, or the Wilcoxon test for related samples. The Bonferroni correction was applied for comparison of three groups as two independent experiments.

## Results

### Patients

The study included 15 patients with a confirmed AX-induced MPE, 9 women and 6 men (mean age, 37.93±17.59 years). The drug involved was AX in 4 cases (26.7%) and AX-clavulanic acid in 11 cases (73.3%) and the mean time interval between drug administration and appearance of symptoms was 33.36±9.3 h. Ten cases (66.7%) were skin test positive to AX and 5 (33.3%) needed a DPT with AX to confirm the diagnosis. The mean time interval between the reaction and this study was 100.85±131.1 months. A control group was composed of 15 sex- and age-matched subjects with no history of DTH and confirmed good tolerance to AX (Table 2).

**Table 2:** Clinical characteristics and results of the allergological work-up in the group of patients studied.

**Pat.**	**Sex**	**Age**	**Drug involved**	**Clinical symptoms**	**Skin test with AX**	**DPT with AX**	**Time interval drug-reaction (hours**)	**Time interval reaction-study (months**)
1	M	23	AX-Clv	MPE	+	ND	36	60
2	M	59	AX-Clv	MPE	-	+	24	60
3	F	17	AX-Clv	MPE	-	+	24	24
4	M	49	AX	MPE	+	ND	24	276
5	F	22	AX-Clv	MPE	+	ND	24	24
6	F	62	AX	MPE	+	ND	36	168
7	F	54	AX-Clv	MPE	+	ND	36	0.16
8	F	61	AX	MPE	+	ND	48	240
9	F	27	AX-Clv	MPE	+	ND	48	12
10	F	58	AX-Clv	MPE	+	ND	24	456
11	M	17	AX-Clv	MPE	-	+	36	0.66
12	F	32	AX	MPE	+	ND	36	48
13	F	41	AX-Clv	MPE	-	+	36	24
14	M	29	AX-Clv	MPE	-	+	24	108
15	M	18	AX-Clv	MPE	+	ND	48	12

F: Female; M: Male; AX: Amoxicillin; AX-Clv: Amoxicillin-Clavulanic acid; MPE maculopapular exanthema; ND: Not done

### TLR expression on moDCs

**Figure 1:**
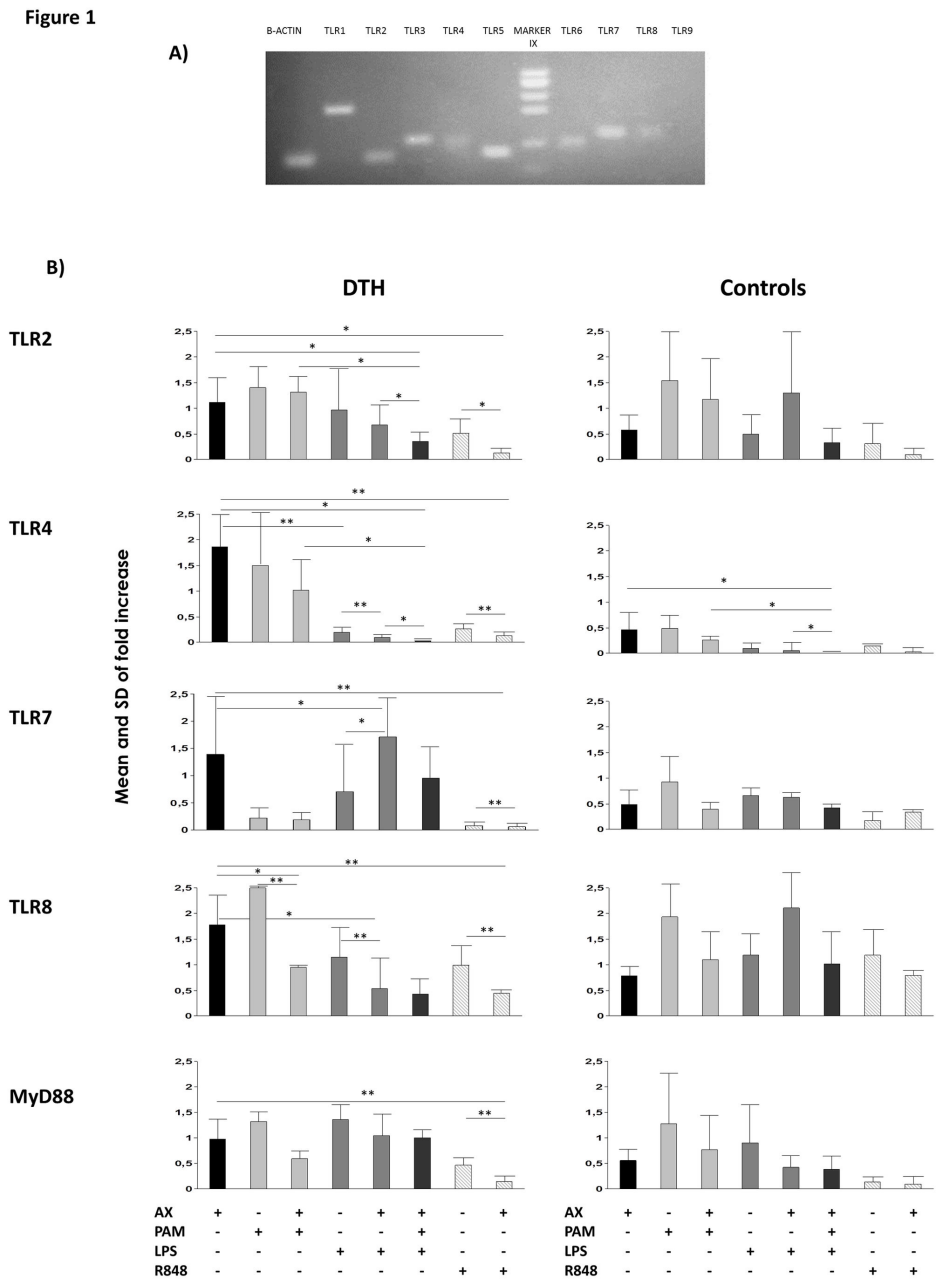
TLR expression on moDC without or with stimuli. **A**) RT-PCR analysis for mRNA expression of different TLRs on imDC from a representative DTH patient. **B**) Bars represent the median fold increase in TLR2, 4, 7 and 8 expression and MyD88 after stimulation with AX, PAM, LPS and R848 alone or in combination on DC from DTH patients (left) and controls (right). *p<0.05, **p<0.01, ***p<0.001.

The expression of different TLRs and the adaptor molecule for signal transduction, MyD88, was assessed by real-time RT-PCR in AX-allergic patients and tolerant controls, detecting expression of TLR1-8 but not TLR9 in imDC (Figure 1A). There were no significant differences (using Mann-Whitney tests) in the TLR expression levels in imDC between both groups (data not shown).

We first compared the effect of AX and different TLR agonists on TLR expression in imDC by the Wilcoxon test (data not shown). We found that AX induced a down-regulation in most TLRs, though only significantly so in controls (p=0.026 for TLR2, 4, 7 and 8 and MyD88) and an overexpression of TLR8 in patients (p=0.034). Regarding the different TLR agonists, PAM significantly increased TLR8 and MyD88 (p=0.011 and 0.034, respectively), and decreased TLR7 (p=0.011) in DTH and TLR4 (p=0.026) in controls. LPS significantly decreased TLR2 and 4 mRNA levels and R848 decreased TLR2, 4, 8, 7 and MyD88 mRNA levels in both DTH and controls.

We then analyzed the synergistic effect of AX and the different TLR agonists in DCs in DTH and in controls by the Wilcoxon test. Differences were considered significant when AX+TLR ligands induced significant changes compared to each stimuli (AX or TLR ligand) alone (Figure 1B). We found that AX+PAM induced a significant down-regulation of TLR8 in DTH, with no changes in controls. For AX+LPS, a significant down-regulation in TLR4 and 8 and an up-regulation in TLR7 was found only in DTH. AX+PAM+LPS showed a significant decrease in TLR2 only in DTH and in TLR4 in both DTH and controls. Finally, AX+R848 produced a down-regulation in TLR2, 4, 7, 8 and MyD88 only in DTH.

### DC maturation and lymphocyte proliferation

**Figure 2:**
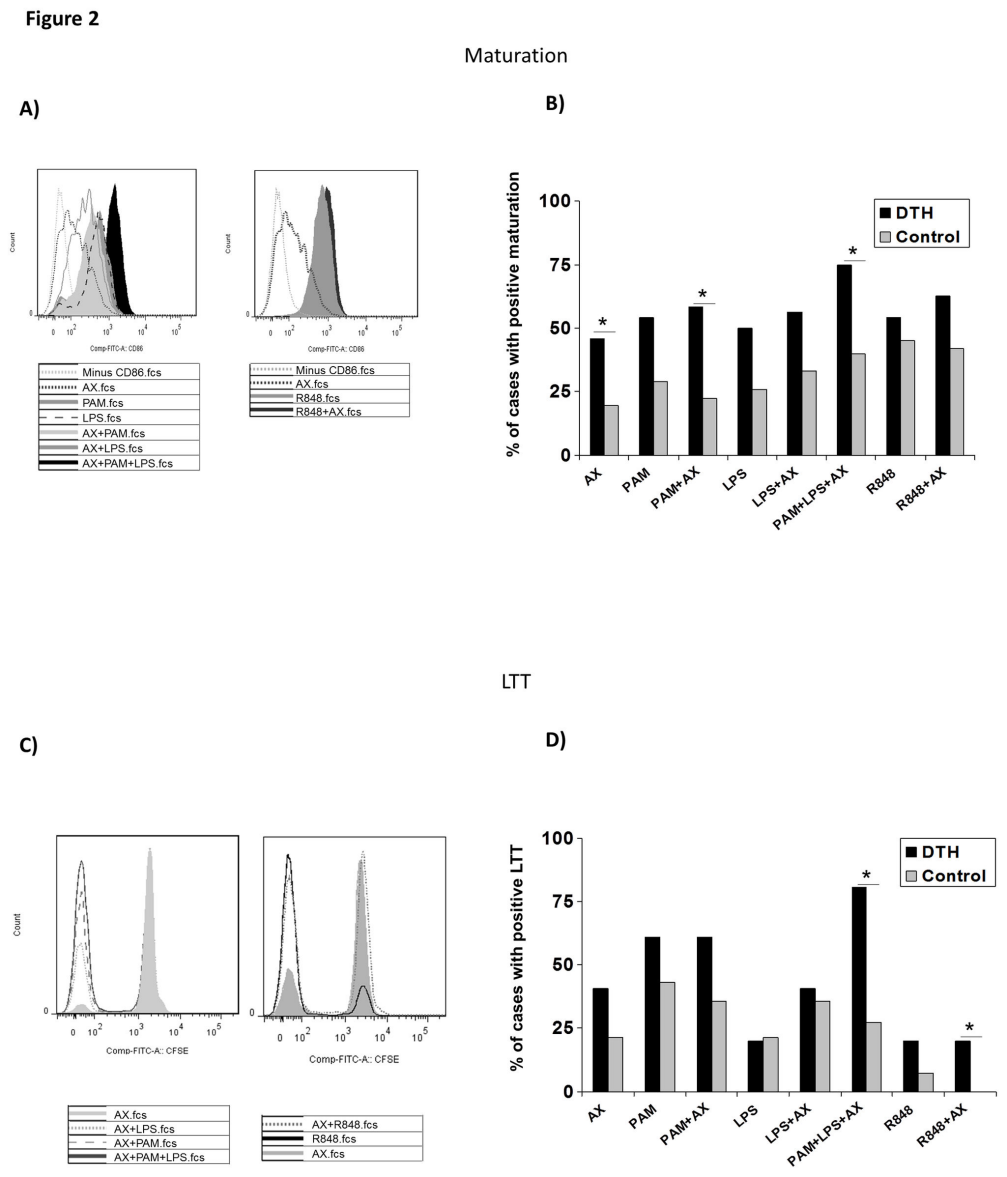
DC maturation and lymphocyte proliferation after different stimuli. **A**) Changes in CD86 expression on moDC after stimulation with AX alone or in combination with PAM, LPS or both (left) and with AX alone or in combination with R848 (right). **B**) Bars represent the percentage of positive cases of DC maturation after stimulation with AX, PAM, LPS and R848 alone or in combination in DC from DTH patients (N=15) (black bars) and controls (N=15) (grey bars). *, p<0.05, represents the significant differences after statistical comparisons (χ^2^ analysis) in the percentage of cases comparing DTH patients to controls. **C**) Changes in CD3^+^ T-lymphocyte proliferation after stimulation with AX alone or in combination with PAM, LPS or both (left) and with AX alone or in combination with R848 (right). Histograms are representative of one DTH patient. **D**) Bars represent the percentage of positive cases of LTT after stimulation with AX, PAM, LPS and R848 alone or in combination in T cells from DTH patients (N=15) (black bars) and controls (N=15) (grey bars). *, p<0.05, represents the significant differences after statistical comparisons (χ^2^ analysis) in the percentage of cases comparing DTH patients to controls.

We evaluated changes in the moDC phenotype after stimulation with AX, PAM, LPS, and R848 alone or in combination in both DTH and controls. Figure 2A shows the changes in CD86 expression on stimulated DCs from a representative DTH patient.

We evaluated 15 patients and 15 controls. The statistical comparisons (χ^2^ analysis) of the percentage of cases with positive DC maturation (Figure 2B) indicated that AX induced a higher percentage in DTH compared to controls (45.8% vs. 19.4%; p=0.035), with no differences after PAM, LPS or R848 stimulation. However, AX+PAM-stimulated moDCs significantly increased the percentage maturation (58.3% in DTH vs. 22.2% in controls; p=0.0085), being even higher when LPS (AX+PAM+LPS) was included (75% in DTH vs. 40% in controls; p=0.036).

Next, we analyzed the differences in T-cell proliferation after stimulation with AX, PAM, LPS, and R848 alone or in combination in DTH (N=15) and controls (N=15). The histogram showing the changes in CFSE^low^ expression lymphocytes after stimulation in a representative DTH patient is shown in Figure 2C. The statistical comparisons (χ^2^ analysis) showed that AX induced a high percentage of positive cases in DTH compared to controls (40.5% vs. 21.4%), with no differences after PAM, LPS or R848 stimulation, and AX+PAM+LPS induced a significant increase in DTH compared to controls (80.7% vs. 27.3%; p=0.001) (Figure 2D).

### Cytokine production by DCs

Analysis with the Wilcoxon test for related samples of the cytokine production by AX-stimulated DCs demonstrated a significant increase in IL-12p70, IL-10 and IL-6 (p=0.046, 0.010 and 0.029, respectively) and a decrease in IL-4 (p=0.046) only in DTH (data not shown).

**Figure 3:**
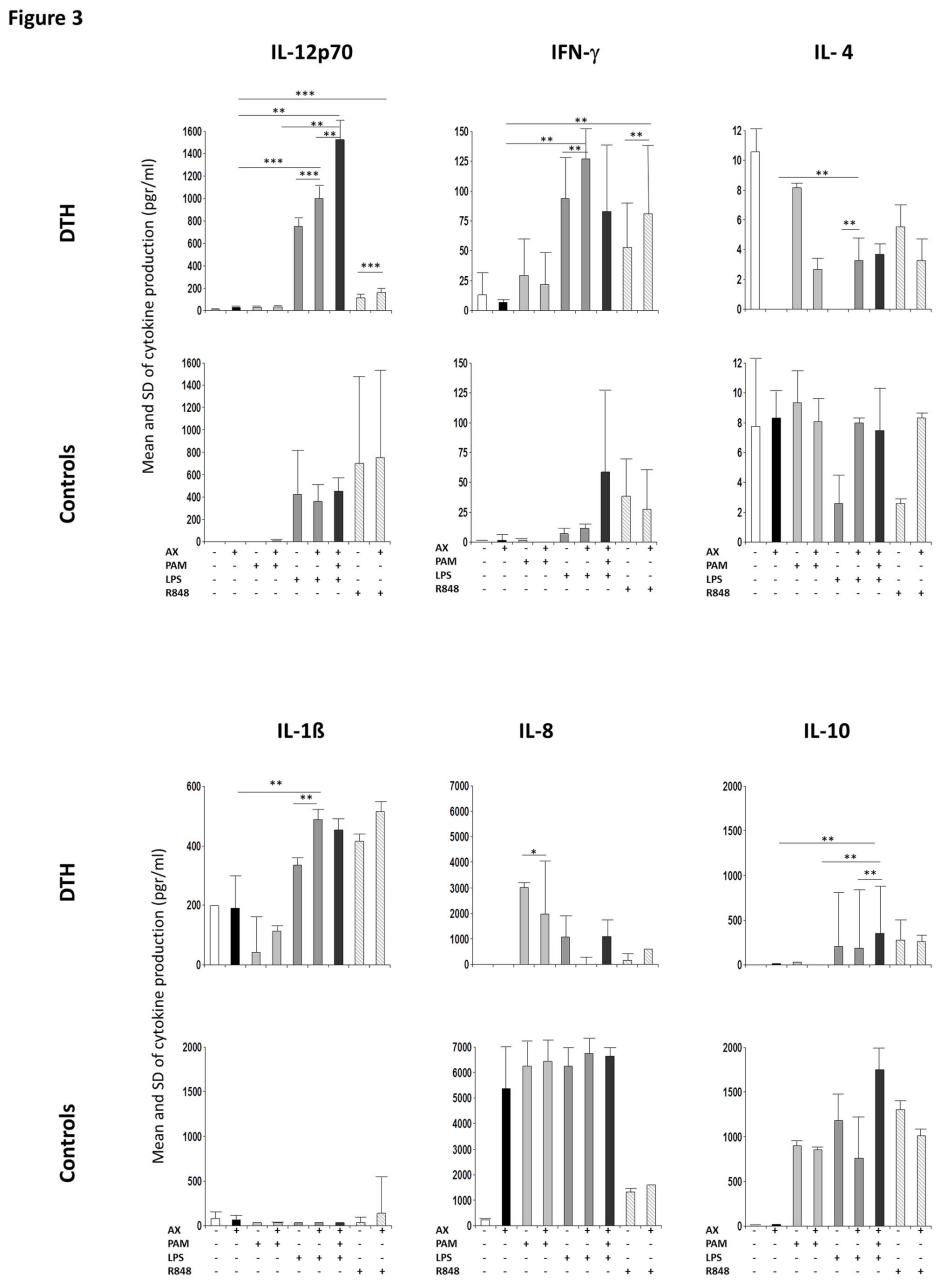
Cytokine production by DC from DTH patients and controls. Bars represent the mean and standard deviations of production of IL-12p70, IFN-γ and IL-4 (top) and IL-1β, IL-8, TNF-α and IL-10 (bottom) after stimulation with AX, PAM, LPS and R848 alone or in combination on DC from patients and controls. *p<0.05, **p<0.01, ***p<0.001.

Following the same statistical analysis with the Bonferroni test, we observed that AX+PAM significantly decreased IL-1β compared to each stimulus alone (p=0.021 and 0.001, respectively) only in DTH. AX+LPS significantly increased IL-12p70 (p=0.001 for both stimuli), IL-4 (p=0.010 for both stimuli) and IL-1β (p=0.002 for both stimuli) only in DTH and IFN-γ in DTH (p=0.010 for both stimuli). Interestingly, we observed that AX impaired IL-8 production in DTH after AX+PAM or AX+LPS stimulation compared to PAM or LPS alone, with the former being significant (p=0.018 and 0.029, respectively) (Figure 3).

AX+PAM+LPS compared to AX, AX+PAM and AX+LPS significantly increased IL-12p70 (p=0.002 for all) and IL-10 (p=0.010 for all) in DTH (Figure 3).

AX+R848 significantly increased IL-12p70 (p<0.001 and p=0.003, respectively) and IFN-γ (p=0.021 for both) in DTH.

### Cytokine production by lymphocytes

**Figure 4:**
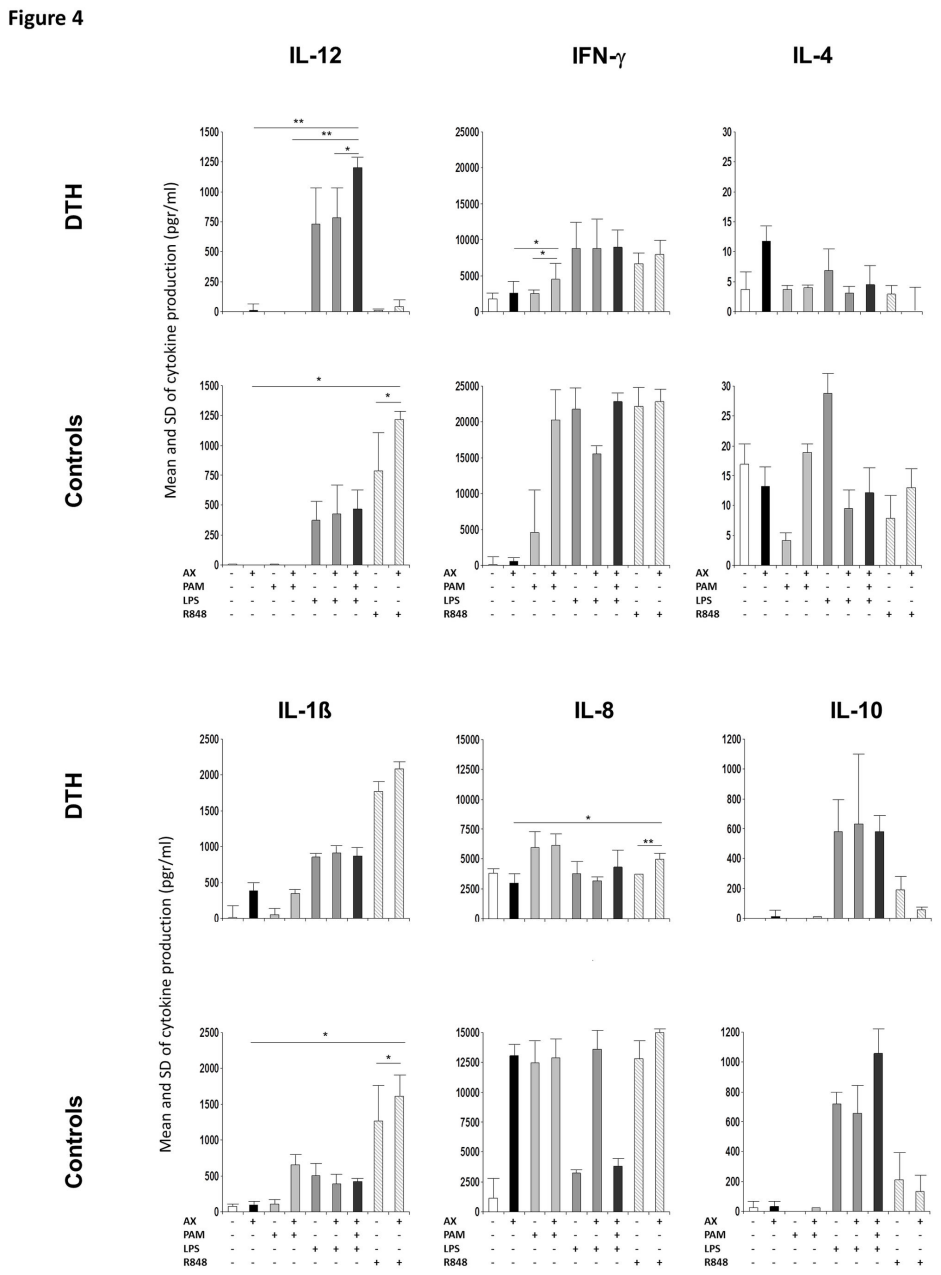
Cytokine production in LTT from DTH patients and controls. Bars represent the mean and standard deviations of production of IL-12p70, IFN-γ and IL-4 (top) and IL-1β, IL-8, TNF-α and IL-10 (bottom) after stimulation with AX, PAM, LPS and R848 alone or in combination of lymphocytes from patients and controls. *p<0.05, **p<0.01, ***p<0.001.

The cytokine production in the supernatants from LTT after stimulation with AX and TLR ligands alone and in combination in DTH and controls was also evaluated. Comparisons with the Wilcoxon test for related samples in non-stimulated cells showed a significant increase for IL-10 in controls compared to DTH (p=0.046). AX induced a significant increase in IFN-γ and IL-1β production (p=0.046, and 0.029, respectively) only in DTH. For IL-8 there was a significant decrease in DTH (p=0.026) and a significant increase in controls (p=0.046) (data not shown). AX+PAM significantly increased IFN-γ in DTH (p=0.012). AX+PAM+LPS significantly increased IL-12p70 compared to AX, AX+PAM and AX+LPS (p=0.011, 0.011 and 0.035, respectively) only in DTH (Figure 4). Finally, AX+R848 significantly increased IL-8 in DTH (p=0.013 and 0.005) in controls and IL-1β in controls (p=0.026 for both).

## Discussion

Low-molecular-weight compounds such as drugs are able to induce DC maturation [11,12], a phenomenon that differs between drug-induced DTH and tolerant subjects [14]. However, AX induces *in vitro* moDC semi-maturation without cytokine production. This may be due to the lack of co-factors like PAMPS present during the infectious disease when the patients take AX and that may help the induction of full DC maturation [9].

In this work we first analyzed the TLR profile on imDC from patients and controls, detecting expression of TLR1-8 but not TLR9, as previously reported [23,24]. Afterwards, we studied the effect of AX on moDC TLR expression, observing a general decrease compared with their respective imDC in both DTH patients and controls, though only significantly so in the latter. Moreover, AX failed to down-regulate the TLR8 mRNA level in DTH. These data suggest that at the basal level, imDCs showed a similar TLR pattern expression in both DTH and controls; however, the interaction of AX with these cells prevented down regulation of the TLRs in DTH. This lack of downregulation detected in patients may affect further DC maturation, as described in polyendocrinopathy candidiasis ectodermal dystrophy [25].

TLRs act synergistically, enhancing DC maturation [26], with TLR2 and TLR4 being the most frequently studied combination. In contact hypersensitivity, two recent studies demonstrated that full DC activation requires not only the allergen but also endogenous danger signals released in the skin and delivered via TLR2/TLR4 signaling pathways [21,27]. The authors showed that only partial DC activation was achieved *in vitro* with the contact allergen alone due to the absence of tissue-derived TLR2/TLR4 mediated danger signals, and this is consistent with our previous results in AX-allergic patients [14]. Thus, triggering of TLRs may be essential to establish an adaptive immunity and Th1 cytokine production [28]. To assess the potential synergistic effects between TLRs and AX, we established different culture conditions and evaluated changes in TLR expression, DC maturation, and lymphocyte proliferation. We found that in combination with TLR ligands, AX induced an important down-regulation of TLR expression only in DTH. These results suggest that the defective downregulation in DTH produced by AX alone can be restored by the addition of microbial ligands interacting with TLRs on DCs and this may be important to mount a specific immune response.

It is interesting to mention the synergistic effect of AX+PAM+LPS, which decreased TLR2 expression only in DTH, and TLR4 in both DTH and controls, indicating that AX may have a more specific effect on TLR2. On the other hand, AX+R848 induced a significant down-regulation of its specific receptors TLR7/8, as well as others (TLR2, 4 and MyD88) only in DTH. All these data indicate the important cross-talk between the different TLR pathways in moDCs, demonstrating that the regulation of TLR expression is controlled by different phenomena such as synergy or cross-tolerance [29,30].

As previously described [19,20], activation via TLRs induces changes in DC maturation. In our work we observed that the downregulation of TLR expression induced by AX in combination with TLR ligands has an effect on DC maturation. In this sense, data showed that the combination of AX with the TLR2 and 4 ligands increased the percentage of DC maturation in DTH (75% of cases) compared to controls (40% of cases) and T-cell proliferation, again being higher in DTH patients compared to controls (80.7% vs. 27.3%).

Next, in order to confirm whether TLR signaling is required for full maturation of AX-stimulated DC, the pattern of cytokine production was analyzed. We found that AX+PAM produced a decrease in the pro-inflammatory cytokine IL-1β with no production of IL-12p70, consistent with previous studies [31,32]. Conversely, AX+LPS significantly increased IL-12p70 and IL-1β production by DCs from DTH. Interestingly, AX+PAM+LPS induced a Th1 profile with higher production of IL-12p70 in DTH patients. In agreement with data on hapten-activated DCs [27], these results strongly support the idea that IL-12p70 production by AX-activated DCs is TLR2 and 4-dependent. Moreover, this combination increased IL-10, involved in regulatory responses, as also described by others [33]. Collectively, these data indicate that AX needs both TLR2 and 4 signaling pathways in order to achieve a stronger Th1 pattern related with the pathophysiology of T-cell mediated drug allergic reactions.

Controversial data have been published about the role of the TLR7/8 ligands in moDC differentiation and maturation [34,35]. Our results indicated that their ligand, R848, in the presence of AX, induced DC maturation with a Th1 profile (IL-12 and IFN-γ) in DTH, indicating that resiquimod promotes the generation of functionally competent AX-stimulated DCs, mimicking the pattern of DTH allergic responses *in vivo*.

IL-8 production was significantly decreased after AX stimulation, even in the presence of TLR agonists, only in DTH. Although we are unaware of the reason for this, it may be related with the pathogenic mechanism involved in MPE, though further studies will be required to determine this.

From these studies we observed that AX is able to interact with DC. However, this interaction by itself does not produce a complete immune response with the absence of cytokine production. We also demonstrated that the presence of TLR ligands, especially the combination of TLR2 and 4 on one hand and TLR7/8 on the other, are required for TLR downregulation and eventually DC maturation and T-cell proliferation with a clear Th1 pattern, which resembles the reaction suffered by the patients at the acute phase.

To summarize, this work shows that in DTH to drugs, such as amoxicillin-induced maculopapular exanthema, the presence of different TLR agonists is critical for the efficient induction of the innate and adaptive immune responses. Further studies need to be carried out to analyze the signaling pathways of each TLR in order to confirm their relevance in the immunological response induced by AX.
